# Early sensory re-education of the hand after peripheral nerve repair based on mirror therapy: a randomized controlled trial

**DOI:** 10.1590/bjpt-rbf.2014.0130

**Published:** 2016-01-19

**Authors:** Mayara H. Paula, Rafael I. Barbosa, Alexandre M. Marcolino, Valéria M. C. Elui, Birgitta Rosén, Marisa C. R. Fonseca

**Affiliations:** 1 Departamento de Biomecânica, Medicina e Reabilitação do Aparelho Locomotor, Faculdade de Medicina de Ribeirão Preto (FMRP), Universidade de São Paulo (USP), Ribeirão Preto, SP, Brazil; 2 Curso de Fisioterapia, Universidade Federal de Santa Catarina (UFSC), Araranguá, SC, Brazil; 3 Curso de Terapia Ocupacional, Faculdade de Medicina de Ribeirão Preto (FMRP), USP, Ribeirão Preto, SP, Brazil; 4 Occupational Therapist, Hand Therapist, Lund University, Sweden

**Keywords:** hand injuries, peripheral nerve injuries, rehabilitation

## Abstract

**BACKGROUND:**

Mirror therapy has been used as an alternative stimulus to feed the somatosensory cortex in an attempt to preserve hand cortical representation with better functional results.

**OBJECTIVE:**

To analyze the short-term functional outcome of an early re-education program using mirror therapy compared to a late classic sensory program for hand nerve repair.

**METHOD:**

This is a randomized controlled trial. We assessed 20 patients with median and ulnar nerve and flexor tendon repair using the Rosen Score combined with the DASH questionnaire. The early phase group using mirror therapy began on the first postoperative week and lasted 5 months. The control group received classic sensory re-education when the protective sensation threshold was restored. All participants received a patient education booklet and were submitted to the modified Duran protocol for flexor tendon repair. The assessments were performed by the same investigator blinded to the allocated treatment. Mann-Whitney Test and Effect Size using Cohen's d score were used for inter-group comparisons at 3 and 6 months after intervention.

**RESULTS:**

The primary outcome (Rosen score) values for the Mirror Therapy group and classic therapy control group after 3 and 6 months were 1.68 (SD=0.5); 1.96 (SD=0.56) and 1.65 (SD=0.52); 1.51 (SD=0.62), respectively. No between-group differences were observed.

**CONCLUSION:**

Although some clinical improvement was observed, mirror therapy was not shown to be more effective than late sensory re-education in an intermediate phase of nerve repair in the hand. Replication is needed to confirm these findings.

## Introduction

Peripheral nerve injuries of the hand have a high incidence^1^ with consequences related to loss of mobility, sensibility, and function^2^. Studies have shown that changes in the cerebral cortex begin within the first minutes after peripheral nerve injury, resulting in an overlap of adjacent cortical areas. These changes occur due to the absence of afferent stimuli in the area of cortical representation of the injured nerve^3^. In order to feed the somatosensory cortex and to preserve the cortical representation of the hand and indirectly facilitate better functional results^4^^,^ 5, early alternative stimuli have been used such as tactile glove or mirror therapy during the initial sensory loss period.

Mirror therapy combined with conventional rehabilitation is a therapeutic alternative in hand rehabilitation to improve range of motion and function in both orthopedic and central nervous system injuries^6^^,^ 7. In addition, a sensory and motor re-education program in the early postoperative phase before any re-innervation hypothetically helps to preserve the somatosensory cortical representation of the hand and to reduce or inhibit the cortical reorganization that would occur without early intervention^8^^-^ 10. However, there is still a need for studies that address the effect of mirror therapy on nerve repair in the hand.

Therefore, we hypothesized that an alternative stimulus during the early phase after nerve repair in the hand would bring better functional results. Therefore, the objective of this study was to analyze the functional outcome of an early re-education program using mirror therapy compared to a late classic sensory re-education program of up to 6 months for median and ulnar nerve repair in the hand.

## Method

### Study design

This study was designed as a parallel randomized controlled trial. This trial report followed the CONSORT guidelines^11^.

### Participants

Participants were of both genders and at least 18 years old. All participants had been submitted to primary repair of the median or ulnar nerves with or without flexor tendon repair and were referred to the study through the Hand Surgery Service of a university hospital from 2009 to 2011. The exclusion criteria were the presence of associated fracture or other chronic metabolic-degenerative diseases related to the peripheral or central nervous system.

### Blinding

Randomization was based on a sequence of random numbers generated by Excel^(r)^. The allocation was concealed by using sealed opaque envelopes. The treatment groups were classic sensory re-education group and mirror therapy group. All participants received the interventions by three physical therapists. The assessments were also blinded and performed by a physical therapist and an occupational therapist. Two other therapists (one physical therapist and one occupational therapist) coordinated the group, the study design, and the outcome assessment.

### Sample size

We calculated that 16 participants were required per group to provide 80% power (Standard Deviation of 0.36 for Rosen score) based on Vordemvenne et al.^12^. The sample size was calculated using Graphpad Statmate (GraphPad Software Inc., San Diego, CA, USA).

### Interventions

All patients with associated flexor tendon repair were submitted to early passive mobilization with the modified Duran protocol^13^ in a dorsal cast for 4 weeks in a semi-flexion wrist position and fingers in extension. Both groups followed a combined standard increasing home program of active and passive exercises aimed at preventing tendon adhesion and improving passive mobility and grip strength. Desensitization and scar management were conducted in the cases where hyperesthesia and adhesion occurred.

### Mirror therapy group

**Figure 1:**
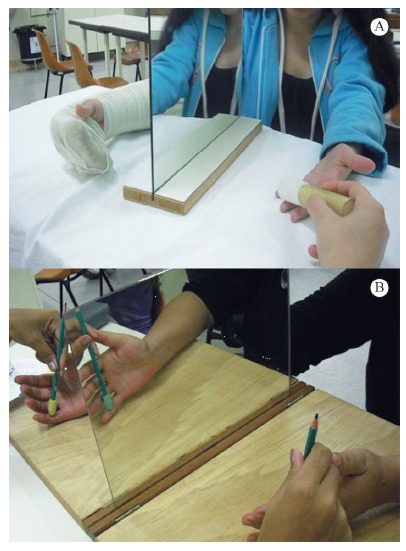
- (A) Mirror placed in front of the patient with tactile stimuli with textures on the first post operative week for the Mirror Therapy group; (B) Mirror placed in front of the patient with tactile stimuli with textures after cast removal for the Mirror Therapy group.

For the mirror therapy group, an early sensory re-education program was initiated in the first postoperative week. A mirror was placed in front of the patient on a table so that the reflected image of the healthy hand looked as if it were the injured hand. Tactile stimuli with several textures and shapes, manipulation of small objects, and active motion on the uninjured hand was performed for 30 minutes a day, 3 times a week, to give the brain the visual illusion of the injured hand. After the cast removal, the procedure was performed bilaterally (Figure 1) for 5 months, in addition to a sensory re-education program. Standard mirror training was performed at home every single day, 30 minutes a day.

### Control group

For the control group, classic sensory re-education was initiated only 3 months after nerve repair to the injured hand when protective sensation returned according to the Semmes Weinstein monofilament test and up to 5 months after surgery. This was performed in the same manner by tactile stimuli with several textures and shapes in a progressive and discriminative way, adding the manipulation of small objects, all included in a similar home program.

All participants received a patient education booklet entitled *"Re-educação sensorial" após reparo nervoso,* translated and adapted from the manual *"Sensory re-education" after nerve repair* by Birgitta Rosén of Lund University, Sweden^14^. It contains an illustrated instruction guide with general information about the trauma and rehabilitation process and how to stimulate hand function in daily life activities at home after nerve repair. The early re-education group received this book on the first week, and the control group received it when sensory re-education started 3 months post nerve repair.

### Key outcome

The Rosen Score was defined as the primary key outcome and the Disability of the Arm, Shoulder and Hand (DASH) questionnaire as a secondary key outcome. The Rosen score^15^^,^ 16, ranging from 0 to 3, was collected 3 and 6 months after surgery. The Rosen score is based on A Model Instrument for Outcome After Nerve Repair^15^, a valid tool that represents a combination of selected instruments clustered in motor domain (motor innervation and grip strength), sensory domain (sensory innervation, tactile gnosis, and finger dexterity), and pain/discomfort domain (hyperesthesia and cold intolerance). In the motor domain, motor innervation was assessed using manual muscle testing and isometric grip strength was tested with a Jamar^(r)^ dynamometer. In the sensory domain, sensory innervation (sensitivity threshold) was measured with Semmes-Weinstein Monofilaments SORRI^(r)17^, tactile gnosis was tested with the Shape-Texture Identification test (STI-test^TM)^ 18 ^,^ 19 and the Weber DiskCriminator^TM^ 20, and finger dexterity was tested with three tasks from the Sollerman grip test (no. 4 - pick up coins, no. 8 - put nuts on bolts, and no. 10 - do up buttons)^21^. In the pain/discomfort domain, hyperesthesia and cold intolerance were assessed by a specific scale.

For function assessment, the DASH outcome measure was used, a self-report questionnaire translated and validated to Brazilian Portuguese^22^. The 30-item instrument evaluates symptoms and physical function on a 5-point Likert scale with total scores ranging from 0 to 100. The higher the score is, the worse the disability.

The present study was approved by the Research Ethics Committee of Hospital das Clínicas de Ribeirão Preto, Universidade de São Paulo (USP), Ribeirão Preto, SP, Brazil, on 03/02/2009 (process no. HCRP 13711/2008). Informed consent was obtained from each subject before participation. This trial was prospectively registered at www.ClinicalTrials.gov (Identifier: NCT01215760).

### Statistical analysis

The statistics analysis was performed using SPSS Statistics^(r)^ software, version 20.0. Shapiro-Wilk test denoted that the Rosen score and the DASH questionnaire deviated from normality. Therefore, the Mann-Whitney test was used for inter-group comparison at 3 and 6 months after intervention (<0.05).

Cohen's d, one of the most standardized measures of the magnitude of an observed effect, was defined for the effect size (ES) calculation using the online ES calculator from the University of Colorado at Colorado Springs: http://www.uccs.edu/~lbecker/. For this study, the benchmarks for effect size were 0.20 = small, 0.50 = moderate, and 0.80 = large^23^.

## Results

**Figure 2:**
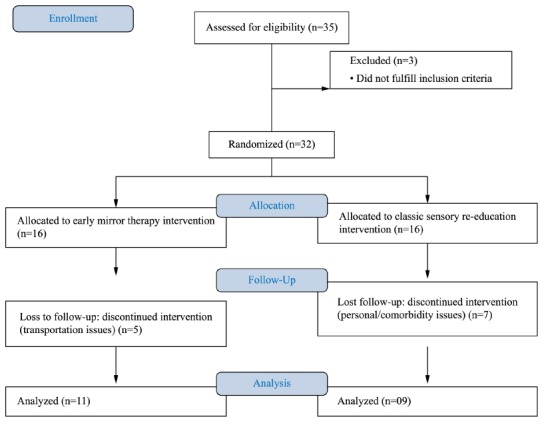
- Flowchart of the group randomization of the RCT

A total of 35 eligible patients volunteered for the trial, but 3 were considered ineligible because of associated comorbidities. Initially, 32 were randomized for Mirror Therapy group (n=16) or Control group (n=16), but 12 discontinued intervention due to transportation/personal issues. The protocol was completed by 20 participants, whose data were analyzed (Figure 2). Most of the participants worked in heavy manual labor occupations (93.3%). Patient demographics are described in Table 1.

**Table 1:** - Demographic data of study participants in each group.

	Control (Classic sensory re-education) (n=09)	Mirror Therapy (n=11)
Age (years)	24.25 (4.8)	29.45 (12.2)
Male Gender (%)	55.5	72.7
Right-handed (%)	77.7	100
Right Affected side (%)	55.5	72.7
Injured Nerve (%)		
Ulnar	44.5	27.3
Median	22.3	54.5
Ulnar and median	33.2	18.2
Location of injury (%)		
Volar aspect of wrist	100	81.8
Palmar aspect of hand/fingers	0	18.2
Type of nerve injury (%)		
Full laceration	100	100
Mechanism of injury (%)		
Glass	88.8	81.8
Saw/knife	0	18.2
Motorcycle accident	11.2	0

Continuous variables are expressed as mean (SD), categorical variables are expressed as percentages.

The mean scores for the Rosen score and DASH questionnaire at 3 and 6 months for each group are summarized in Table 2. No significant differences were observed for DASH and Rosen scores in the between-group comparison. The total Rosen score and its domains for both groups for 3 and 6 months are presented in Table 3.

**Table 2:** - Mean scores (SD and min-max) for DASH and Rosen score for Control classic and Mirror Therapy group after 3 and 6 months.

	Control classic group		Mirror Therapy group		P value for Between-group differences	
	3 months	6 months	3 months	6 months	3 months	6 months
DASH	38.62±27.66 (3.33-100)	27.84±23.35 (2.4-70)	24.25±19.38 (5.8-58.33)	20.34±17.68 (5.8-60)	0.20	0.77
Rosen score	1.65±0.52 (1.11-2.51)	1.51±0.62 (0.71-2.76)	1.68±0.50 (0.78-2.34)	1.96±0.56 (1.00-2.80)	0.82	0.11

**Table 3:** - Protocol for documentation of hand function after nerve repair - total Rosen-Score15,16 filled with mean scores of domains at 3 and 6 months after nerve repair for control and mirror groups.

Domain	Subtests		Mirror group 3 months	Control group 6 months	Mirror group 3 months	Control group 6 months
Sensory Domain						
Innervation	Semmes-Weinstein Monofilament Test, mini-kit		0.61	0.48	0.81	0.76
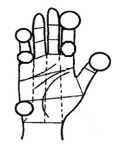	0 = not testable	Results: 0-15				
	1 = filament 6.65 (magenta)					
	2 = filament 4.56 (red 4g)					
	3 = filament 4.31 (violet 2.0g)					
	4 = filament 3.61 (blue 0.2g)	Normal median: 15				
	5 = filament 2.83 (green 0.05g)	Normal ulnar: 15				
Tactile gnosis	2PD (finger II and V)	Results: 0-3	0.26	0.20	0.43	0.34
	0 = ≥16 mm					
	1 = 11-15 mm					
	2 = 6-10 mm					
	3≤5 mm	Normal: 3				
	STI test (fingers II and V)	Results: 0-6 Normal: 6	0.15	0.12	0.23	0.14
Finger dexterity	Sollerman test	Results: 0-12	0.34	0.62	0.53	0.67
	(tasks 4, 8, and 10)	Normal: 12				
Mean score for Sensory Domain:			0.34	0.35	0.50	0.49
Motor Domain						
Innervation	Manual muscle test 0-5		0.57	0.41	0.70	0.50
	Median: palmar abduction	Result median: 0-5				
	Ulnar: abduction fingers II/V	Result ulnar: 0-15				
	Adduction finger V	Normal median: 5				
		Normal ulnar: 15				
Grip strength	Jamar dynamometer in second position Normal: Result uninjured hand Mean of three trials, right and left		0.39	0.34	0.62	0.44
Mean score for Motor Domain:			0.48	0.37	0.66	0.47
Pain/discomfort Domain						
Cold intolerance/ hyperesthesia	Patient’s estimation of perceived problems					
	0 = Hinders function	Result: 0-3				
	1 = Disturbing					
	2 = Moderate					
	3 = None/minor	Normal: 3				
Mean score for Pain/Discomfort Domain:			0.86	0.86	0.80	0.65
TOTAL SCORE (Sensory + Motor + Pain/Discomfort):			1.68	1.58	1.96	1.61

## Discussion

Our clinical trial did not demonstrate any advantages of Mirror Therapy over classic re-education. This early approach using visual illusion by mirror therapy was implemented in a phase before any re-innervation had occurred and assessed by the Rosen score through its subtests related to the motor, sensory, and pain/discomfort domains. For the classic re-education control group, the Rosen score was even lower for the sensory and pain/discomfort domains after 6 months (Table 3). Rosén and Lundborg^24^ also found poor results for this domain after 6 months postoperative. The pain/discomfort domain includes cold sensitivity and hypersensitivity items. Cold intolerance item is often an issue following nerve injuries. To increase validity, a reference score for tropical climate conditions should be developed in comparison to countries with a colder climate.

Outcome measurements following nerve repair provide information about the patients' sensorimotor deficits and function^25^^,^ 26. In a systematic review, Jerosch-Herold^27^ described a few of sensory tests for nerve repair with sufficient evidence of reliability, validity, and responsiveness. These tests range from a numerical grading scale for peripheral nerve function^28^ to a model instrument for documentation after nerve repair that includes sensory, motor, and pain/discomfort outcomes, which together constitute the Rosen score^15^^,^ 16 used in the present study. A reference interval^24^ for recovery after median and ulnar nerve repair in adults can be easily used in clinical practice and in combination with the DASH questionnaire for functional assessment following nerve injury and repair^27^.

Sensory re-education is well established as a therapeutic method for nerve repair^29^, however it is not advocated by most therapists^30^ as part of early phase retraining with sensory substitution when no re-innervation has occurred. Although few studies have addressed this approach, interest has improved in the area. A study^31^ reviewed 67 cases treated with classic sensory re-education and found good recovery of perception of touch but poor ability to use this capacity to identify and discriminate touch (tactile gnosis) 3 months after nerve repair in the hand. In a recent systematic review of clinical trials on the effects of re-education programs in functional hand sensibility after median and ulnar repair, the authors found just one study that investigated early phase retraining using the Model Instrument with moderate evidence in tactile gnosis but not in the composite score^32^. In a multi-center randomized controlled trial, adults with median or ulnar nerve repair at the distal forearm were randomized to mirror visual feedback intervention, and at 6 months, discriminative touch was significantly better in the early intervention group^33^, corroborating the data of the present study.

Cortical plasticity is an intrinsic property of the central nervous system. Although cortical plasticity following nerve injury is still not fully understood, peripheral nerve injuries are known to result in "black holes" in somatosensory cortex in primates with changes in representation after a few weeks, depending on external input stimulation^34^. Methods such as mirror therapy^9^^,^ 10 or sensory glove^5^^,^ 8 allow early sensory re-education before re-innervation is detected. These concepts aim to facilitate peripheral sensory integration with the cortex area and promote interaction between tactile, visual, and auditory stimuli. Therefore, they are important tools to optimize sensory re-education strategies and maximize the preservation of the hand's cortical map representation in the early phase following injury. In addition to these new rehabilitation and surgical concepts, there is still not a single technique that ensures full recovery of tactile discrimination of the hand in adults following peripheral nerve injury^16^. Therefore, early strategies for sensory re-education, such as the mirror therapy used in this study, could be adopted into the sensory and functional rehabilitation process after nerve repair.

Although our sample was small, it was representative of the profile of this type of injury with complete transection of the median and ulnar nerves^1^^,^ 2 ^,^ 35 ^,^ 36. Our sample was mostly young and male who suffered cuts from glass. The early sensory re-education group had a predominance of median nerve injuries, which could have brought better sensory and motor results and consequent superior short-term functional outcomes in comparison to ulnar injuries^12^.

A limitation of this study is that the assessment was performed only during the first six months following the nerve repair. Regeneration of the repaired nerve and recovery of function can take several years, and improvements, especially in discriminative touch, have been documented up to at least five years after the repair^37^. A longer follow-up, when touch threshold at fingertip level would be lower, might show more distinct results after early sensory re-education. Other limitations of our study include a high loss to follow-up and absence of intention-to-treat analysis. These two methodological issues are likely to overestimate our results^38^^,^ 39. Therefore, caution is needed when interpreting our results.

## Conclusion

Mirror therapy combined with an early re-education program was not shown to be more effective than late sensory re-education in the intermediate phase of nerve repair to the hand, based on the functional outcome Rosen Score after traumatic injury of the median and ulnar nerves in adults. Replication is necessary to confirm these findings.
